# Single-Cell Transcriptomics: Current Methods and Challenges in Data Acquisition and Analysis

**DOI:** 10.3389/fnins.2021.591122

**Published:** 2021-04-22

**Authors:** Asif Adil, Vijay Kumar, Arif Tasleem Jan, Mohammed Asger

**Affiliations:** ^1^Department of Computer Sciences, Baba Ghulam Shah Badshah University, Rajouri, India; ^2^Department of Biotechnology, Yeungnam University, Gyeongsan, South Korea; ^3^School of Biosciences and Biotechnology, Baba Ghulam Shah Badshah University, Rajouri, India

**Keywords:** single-cell transcriptomics, Sc-RNA-seq, big data, single-cell big data, normalization, single-cell analysis, downstream analysis

## Abstract

Rapid cost drops and advancements in next-generation sequencing have made profiling of cells at individual level a conventional practice in scientific laboratories worldwide. Single-cell transcriptomics [single-cell RNA sequencing (SC-RNA-seq)] has an immense potential of uncovering the novel basis of human life. The well-known heterogeneity of cells at the individual level can be better studied by single-cell transcriptomics. Proper downstream analysis of this data will provide new insights into the scientific communities. However, due to low starting materials, the SC-RNA-seq data face various computational challenges: normalization, differential gene expression analysis, dimensionality reduction, etc. Additionally, new methods like 10× Chromium can profile millions of cells in parallel, which creates a considerable amount of data. Thus, single-cell data handling is another big challenge. This paper reviews the single-cell sequencing methods, library preparation, and data generation. We highlight some of the main computational challenges that require to be addressed by introducing new bioinformatics algorithms and tools for analysis. We also show single-cell transcriptomics data as a big data problem.

## Introduction

The human body exhibits a diverse range of cells that undergo transit from one state to another in life (development, disease, and regeneration). Though derived from the same zygote, the cell, with its types and states, is greatly influenced by the internal processes and external factors (Song et al., 2019). In its progression through proliferation and the differentiation states to generate multiple cell types for organ formation, complex heterogeneities in the cellular architecture are observed. The cellular heterogeneity in terms of morphology, function, and gene expression profiles lie between various tissues, but has also been observed among the same cell types that allow them to perform different roles. Dysregulation in any particular cell type (irrespective of tissues, organs, and organ-system) influences the entire system that progresses to disorders and even severe diseases like cancer (Macaulay et al., 2017).

Recent technological advancements have enabled biologists to profile *cells at individual levels* on a variety of omics layers (genomes, transcriptomes, epigenomes, and proteomes) (Hu et al., 2016); among these, single cell (SC) transcriptomics is widely studied. The cells of a human body, being heterogeneous, often show a drastic variation at the individual level (Wang and Bodovitz, 2010; Xin et al., 2016). The SC experiments were found much conclusive compared with bulk cell sequencing that involves sequencing in bulk (assuming cells of a particular type are identical) and estimating an average of expressions. The SC transcriptomics was awarded as method of the year by *Nature* in 2013 (Xue et al., 2015). With the advent of next-generation sequencing, it becomes possible to develop sequencing methods to probe the dynamics of the genome and variations thereof. Of them, RNA sequencing (RNA-seq)-mediated transcriptomic profiling revealed information of novel RNA species that deepened our understanding of the transcriptome dynamics (Tang et al., 2009; Wang et al., 2009; Ozsolak and Milos, 2011). Lately, these sequencing approaches have been extended to study intra-population heterogeneity of SCs (Wills et al., 2013), whereby it enabled the study of cell fates, their transition to different subtypes, and the dynamics of gene expression masked in bulk population studies (Altschuler and Wu, 2010; Trapnell et al., 2014). Compared with bulk sequencing, where libraries are prepared from thousands of cells, libraries for single-cell RNA sequencing (SC-RNA-seq) are cell-specific towards investigating cellular functionalities of DNA and RNA in different cellular subsets (Gross et al., 2015; Xue et al., 2015). Though SC-RNA-seq has revealed novel findings in different cellular backgrounds, it poses specific challenges: Pre-processing of the SC-RNA-seq data is majorly different from bulk RNA-seq, stricter protocols for library preparation and low starting material. Another challenge is the lack of analytical approaches required to accommodate large datasets generated during SC-RNA-seq experiments. Keeping this in view, we investigated the methods adopted in SC experiments, sequencing approaches, and challenges thereof, as part of realizing the goal of precision medicine.

## Single-Cell RNA Sequence Profiling Techniques

With the first report in 2009, a surge in the SC transcriptomics methods capable of sequencing millions of cells with great accuracy and viability in a short span of time was observed (Tang et al., 2009). These methods are generally different from each other in terms of cell isolation methods, cell lysis procedure, amplification process, cDNA generation, transcript coverage, and Unique Molecular Identifier (UMI) tagging (at either 3′ end or 5′ end). The most critical distinction in the SC-RNA profiling techniques is that some provide full-length transcript coverage and some only partially sequence from either 3′ end or 5′ end of the transcript (Chen et al., 2019). Table 1 highlights widely used SC-RNA profiling methods in terms of different properties.

**TABLE 1:** Current SC-RNA-seq profiling techniques, based on transcript coverage and UMI insertion possibility.

**Method**	**Length of transcript**	**UMI insertion possibility**	**References**
ScNaUmi-seq	Full length	Yes	Lebrigand et al., 2020
MATQ-seq	Full length	Yes	Sheng and Zong, 2019
10× Chromium	3′ end	Yes	Zheng et al., 2017
CEL-seq2	3′ end	Yes	Hashimshony et al., 2016
Drop-seq	3′ end	Yes	Macosko et al., 2015
InDrop	3′ end	Yes	Klein et al., 2015
Smart-seq2	Full length	No	Picelli et al., 2014
STRT-seq	5′ end	Yes	Islam et al., 2014
MARS-seq	3′ end	Yes	Jaitin et al., 2014
Smart-seq	Full length	No	Ramskold et al., 2013

*SC-RNA-seq, single-cell RNA sequencing; UMI, Unique Molecular Identifier.*

## Optimal Methodology of Single-Cell Transcriptomics

Of the various sequencing platforms, Drop-seq, InDrop, and 10× Chromium are well-known platforms for sequencing hundreds and thousands of cells in an unbiased manner (Kulkarni et al., 2019). In SC transcriptomics, each cell needs to be isolated from its originating tissue. The Droplet-based techniques, which at the core use microfluidics to attach cells with beads containing a unique barcode, are widely incorporated to separate cells. The performance criteria for isolation methods are based on three parameters: throughput, purity, and recovery (Tomlinson et al., 2013; Gross et al., 2015). *Throughput* indicates the number of cells that can be isolated per unit time, *purity* refers to the number of cells collected after separation from tissue, and *recovery* is the final amount of the target cells, in hand, after separation. The morphological complexity of cells like those of the central nervous system (CNS) makes the separation process a little challenging. The segregation process exposes them to specific environmental, chemical, and harsh dissociation steps that often bias data analysis (Kulkarni et al., 2019). The dissociation of intact cells from a frozen postmortem tissue is also challenging, as cell membranes are prone to damage from mechanical and physical stresses as part of the freeze–thaw process (McGann et al., 1988). Though each cell separation methods currently in use shows an advantage different for the above three parameters, it becomes imperative to select a well-suited method for the isolation of a cell. The current methodology of cell separation is broadly categorized into two groups based on (1) cellular properties like cell density, cell shape, cell size, etc., and (2) biological characteristics of a cell that comprises affinity methods (Tomlinson et al., 2013). Tables 2, 3 show some of the widely used methods concerning the operational mode, throughput, advantages, and disadvantages.

**TABLE 2:** Commonly used methods for cell isolation based on biological characteristics.

**Technique**	**Mode of operation**	**Throughput**	**Advantages**	**Disadvantages**	**References**
Fluorescence-activated cell sorting	Automatic	High	High rate of rare cell sorting, high purity	Cost-intensive, high skills required	Herzenberg et al., 2002; Gross et al., 2015
Magnetic-activated cell separation	Automatic	High	High purity, cost-efficient	Cell capture is non-specific	Schmitz et al., 1994; Welzel et al., 2015

**TABLE 3:** Commonly used methods for cell isolation on the bases of physical characteristics.

**Technique**	**Mode of operation**	**Throughput**	**Advantages**	**Disadvantages**	**References**
Microfluidic cell separation	Automatic	High	Works with low starting materials, amplification integration	High skills required, dissociated cells	Wyatt Shields et al., 2015
Micromanipulation manual cell picking	Manual	Low	More control over cell, live and intact cell separation	Laborious, high skills needed	Citri et al., 2012
Laser-capture microdissection	Manual	Low	Undamaged live cell capture, highly advanced	Too complex to operate, threat of contamination by neighboring cells	Espina et al., 2006
Density gradient centrifugation	Manual	Low	Cost-efficient	Too slow and laborious, low yield	Beakke, 1951

**FIGURE 1:**
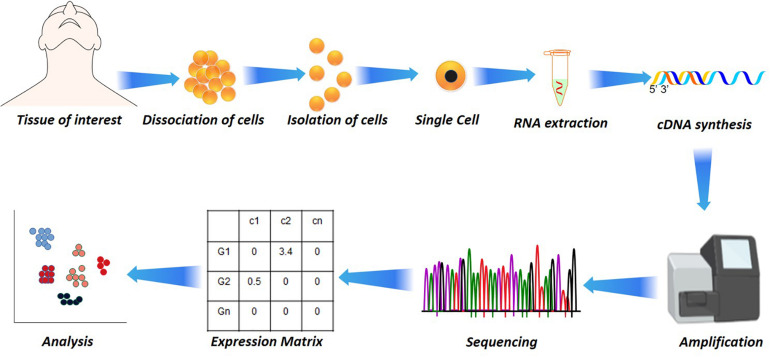
Single-cell analysis in disease and health. Starting from the dissociation of target cells from the target tissue/organ, their isolation based on fluorescence-activated cell sorting (FACS) or other microfluidic techniques to RNA extraction. The RNA extraction is followed by cDNA synthesis by reverse transcriptase, followed by amplification and sequencing. From the sequencing, the reads are aligned and subjected to quantification that results in a quantification matrix or Gene Expression Matrix.

Though high-throughput SC-RNA approaches such as 10× Chromium allows analysis of cells in an unbiased manner, it lacks in providing an in-depth information on sequence diversity, splicing, and chimeric transcripts generated in the process (Lebrigand et al., 2020). The problem is overcome by performing Nanopore long-read sequencing [using a cell barcode (cellBC) assignment to long reads] to obtain a full-length sequence corresponding to the 10× Chromium system’s data. As SC library preparation requires robust amplification, chimeric cDNA generation and amplification bias issues are currently addressed by employing a 3′ or 5′ end tag-based approach (Trombetta et al., 2015; Natarajan et al., 2019). The sequence length method determines the quality of alignment across the total length of a gene, while tag-based methods integrate UMIs at either 3′ end or 5′ end of the transcript (Kivioja et al., 2012; Smith et al., 2017; Sena et al., 2018). The UMI addition makes it easier to identify and quantify the individual transcripts by eliminating PCR artifacts and minimizes false annotation of PCR-generated chimeric cDNAs as novel transcripts. The full length-based methodology provides an all-inclusive coverage of the reads, yet they contribute a bias for long genes, as the genes with shorter length are often missed (Phipson et al., 2017). Additionally, the higher sequencing error rate of long-read sequencers and UMI problems account for a serious issue pertaining to these platforms (Gupta et al., 2018; Lebrigand et al., 2020; Volden and Vollmers, 2020). Despite this, the Tag-based methods have shown a fair dominance in SC-RNA library preparation for quantifying the transcripts in SC analysis when cell number is large (Figure 1).

## Quantification of Expression and Quality Control

Like bulk RNA-seq, the transcripts in SC-RNA are sequenced into reads that generate the raw fastq data. The quality of the sequence reads generated in a sequencing method is considered an important quality indicator of SC-RNA-seq data. As the alignment of the transcript reads for SC-RNA-seq is same as bulk RNA-seq, the methods and tools used for the gene or transcript quantification for bulk RNA-seq can also be used for quantifying transcripts generated by SC-RNA-seq (Li and Homer, 2010; Fonseca et al., 2012). HISAT2 (Kim et al., 2019), TopHat2 (Kim et al., 2013), and STAR (Dobin et al., 2013) are currently the most popular alignment tools, which can map billions of reads to a reference transcriptome with greater accuracy and high speed. Transcriptome reconstruction can be either *de novo* (for samples lacking reference genome) or reference based, also called genome-guided assembly (Chen et al., 2011). However, the former technique sometimes lacks accuracy in comparison with the reference-based assembly approach (Garber et al., 2011). For SC-RNA-seq methods that generate data on a whole-transcriptome basis, Smart-seq2 (Picelli et al., 2014) and MATQ-seq (Sheng and Zong, 2019) use Cufflinks, RSEM, Stringtie, etc., for the quantification of transcripts, while methods that incorporate the 3′ end UMI tagging [like Drop-seq (Macosko et al., 2015), InDrop (Klein et al., 2015), MARS-seq (Jaitin et al., 2014), etc.] require specific algorithms to generate the expression count for the transcript. Another efficient tool for the UMI-based methods was developed by Huang and Sanguinetti (2017) for calculating the expression count of SCs accurately. Table 4 provides information about the current tools for read alignment and expression quantification. The SC-RNA-seq exhibits certain limitations, which results in higher technical noise (Kolodziejczyk et al., 2015). In SC-RNA-seq data, many transcripts appear to be lost during reverse transcription due to the small number and low capture efficiency of RNA molecules in SCs (Saliba et al., 2014). Consequently, in one cell, some transcripts are highly expressed but are missing in another cell. This pattern is described as a “dropout” event. It has been reported that even the most sensitive protocol for SC-RNA-seq fails to detect some of the transcripts as part of Dropout events (Haque et al., 2017). When the cells are dissociated or isolated, a certain number of cells become dead or get destroyed. The SC-RNA-seq methods generate low-quality data from these cells (Ilicic et al., 2016). After alignment and quantification of the transcripts, the quality control check of cells is necessary to remove low-quality cells for an accurate downstream analysis.

**TABLE 4:** Widely used tools for read alignment and expression quantification.

**Tool**	**Function**	**Feature**	**URL**	**References**
Salmon	Expression quantification	k-mer-based read quantification	https://combine-lab.github.io/salmon/	Patro et al., 2017
Kallisto	Expression quantification	Pseudoalignment-based rapid read determination	https://pachterlab.github.io/kallisto/	Bray et al., 2016
StringTIe	Expression quantification	Alignment based, splice aware	https://ccb.jhu.edu/software/stringtie/	Pertea et al., 2015
HISAT2	Read alignment	Alignment based, splice aware	https://daehwankimlab.github.io/hisat2/	Sirén et al., 2014
Sailfish	Expression quantification	k-mer-based read quantification	http://www.cs.cmu.edu/~ckingsf/software/sailfish/	Patro et al., 2014
RNA-Skim	Expression quantification	*Sig-mer* (a type of k-mer)-based read quantification of transcripts	http://www.csbio.unc.edu/rs/	Zhang and Wang, 2014
TopHat2	Read alignment	Alignment based, splice aware	https://ccb.jhu.edu/software/tophat/index.shtml	Kim et al., 2013
STAR	Read alignment	Alignment based, splice aware	https://github.com/alexdobin/STAR	Dobin et al., 2013
Bowtie	Read alignment	Maintains quality threshold, hence less no. of mismatches	http://bowtie-bio.sourceforge.net/index.shtml	Langmead et al., 2009
Cufflinks	Expression quantification	Alignment based, splice aware	https://github.com/cole-trapnell-lab/cufflinks	Trapnell et al., 2010

## Challenges Impeding Single-Cell RNA sequence Data Analysis

Though SC-RNA-seq has deepened our understanding of the cellular heterogeneity and molecular basis of life, it is impeded by several technical and computational challenges. The foremost among them is that its datasets exhibit a considerable amount of noise attributed to meager starting materials that often causes faulty downstream analysis and erroneous results (Brennecke et al., 2013). The SC-RNA-seq data analysis is performed as subtle execution in computational steps; read alignment, expression count generation, cell quality control, normalizing the data, and then further downstream analysis including SC clustering, differential gene expression (DGE), pseudo-temporal analysis, etc. In addition to low starting materials, the technical noise in the datasets is contributed by various factors, like batch effects (Haghverdi et al., 2018) and the low capture efficiency of protocols (Hwang et al., 2018). A few of the analytical steps, including read alignment and generation of count matrix, can be resolved using already available computational methods designed for bulk RNA-seq. However, data processing tasks like normalization, DGE analysis, cell imputation, and dimensionality reduction, etc., call for the development of novel computational techniques, algorithms, and tools for smooth execution of SC-RNA-seq data analysis. The nature of the challenges that SC-RNA-seq data possess, including big data problem (Costa, 2012; Yu and Lin, 2016; Angerer et al., 2017; He et al., 2017), is highlighted in the following subsections:

## Normalization

In SC-RNA-seq, coverage of sequences between the libraries exhibit systematic differences from experimental procedures, dropout events, depth of the sequencing, and other technical effects (Stegle et al., 2015). These differences must be corrected by normalizing the data such that there is no interference in the comparison of the gene expression between cells. Being crucial, normalization of the SC-RNA-seq datasets eventually leads to lucid downstream analysis, including identifying different cell subsets and revealing differential expression of genes. In bulk RNA-seq, expression counts from various libraries are usually normalized by computing the fragments per kilobase of transcript counts of per million mapped fragments (FPKM) (Mortazavi et al., 2008), transcripts per million (TPM) (Li and Dewey, 2011), reads per kilobase of transcripts per million mapped reads (RPKM), upper quartile (UQ) (Bullard et al., 2010), DESeq (Love et al., 2014), removed unwanted variation (RUV) (Risso et al., 2014), and Gamma regression model (Ding et al., 2015). Generally, there are two types of normalization: (1) normalization of data within the sample, and (2) normalization of the data between the sample (Vallejos et al., 2015, 2017). In the former, FPKM/RPKM or TPM are used to exclude gene-specific biases (Vallejos et al., 2017) such as guanine–cytosine (GC) content and gene length, while in the latter, the normalization method tunes the sample-specific differences such as sequencing depth and capture efficiency. While ignoring the underlying stochasticity, normalization generates a relative expression estimate (Stegle et al., 2015), assuming the overall processed RNA per sample is equal (AlJanahi et al., 2018; Olsen and Baryawno, 2018). The bulk-based strategies for normalization have been reported unsuitable for SC-RNA-seq datasets because the datasets are highly zero-inflated and have higher technical noise. Multiple methods have been developed for normalizing the SC-RNA-seq data (Vallejos et al., 2015; Lun et al., 2016; Sengupta et al., 2016; Bacher et al., 2017; Yip et al., 2017). However, *O*(*nlogn*) is considered more efficient than others in performing normalization of SC-RNA-seq data (Yip et al., 2017).

### Dimensionality Reduction

High dimensionality is yet another challenge that SC-RNA-seq data present. Owing to the data coming from cells showing high dimensions, i.e., a large number of genes, it is necessary to reduce (while optimally preserving the critical properties) the set of random variables and work with the principle variables which describe the data profoundly (Andrews and Hemberg, 2019). The two most frequently used methods for dimensionality reduction are principal component analysis (PCA) (Van Der Maaten et al., 2009) and T-distribution stochastic neighbor embedding (t-SNE) (Van Der Maaten and Hinton, 2008; Kobak and Berens, 2019). PCA uses a linear process to transform a set of variables (possibly correlated) into an uncorrelated variable known as a principal component, while t-SNE is a non-linear probability distribution-based approach. Both PCA and t-SNE methods of dimensionality reduction have certain limitations (Chen et al., 2019); based on the assumption that approximately all the data are distributed normally, PCA does not effectively amount to the underlying complexities in the structure of SC-RNA-seq data, and t-SNE has a larger time complexity reaching *O*(*n*^2^) (Pezzotti et al., 2017). The most recent algorithm employed for dimensionality reduction “UMAP” (Uniform Manifold Approximation and Projection) (McInnes et al., 2018; Becht et al., 2019) outperforms PCA and t-SNE for SC-RNA-seq in terms of high reproducibility and meaningful organization of cells (Becht et al., 2018). UMAP is a non-linear graph-based algorithm that tends to identify the closest neighbors of a data point and assigns them a larger weight, thereby preserving the topological structure of the data. The idea is to project a low-dimensional representation of the data while preserving the nearest neighbours of an individual data point (i.e., cells). This helps to group more closely related neighbours and partly conserves the relation of points in the “long-range” using the intermediate data points. Although the interpretation of the distances in a reduced space becomes difficult, UMAP has been largely able to uncover the elusive features of the data. UMAP is computationally faster than t-SNE, preserves the global structure, and maintains the continuity of cell subsets (Becht et al., 2018). At the core, UMAP assumes the subsistence of a “manifold structure” in the data. This assumption makes it find the manifolds in the noise of data. Since SC-RNA-seq suffers from a significant amount of noise, it is necessary to consider it before applying UMAP (McInnes et al., 2018).

Another method to perform dimensionality reduction is the linear discriminant analysis (LDA). LDA is a supervised dimensionality reduction method that tends to maximize the separability between the predetermined classes, using the covariance of “between-class” and “within-class.” It first calculates the mean of the distances between the classes and then the mean of distances within the classes. The goal is to find a projection to maximize the ratio of between-class variability to the lower within-class variability (Tharwat et al., 2017; Qiao and Meister, 2020).

The SC-RNA-seq exhibits potential challenges similar to text mining, such as polysemy and synonymy, noise, and sparsity. Recently, a popular text mining technique, latent semantic analysis (LSA), has been used in SC-RNA-seq dimensionality reduction (Cheng et al., 2019). LSA at core uses a linear algebra-based method, called singular value decomposition (SVD), to cluster the semantically similar terms. SVD approximates a low-rank matrix to the given cell-gene matrix, such that the dimensions of the new matrix are much less than the original. This approximation is made by taking a combined product of the matrices of left-singular vector, right-singular vector, and the diagonal singular values.

### Differential Gene Expression Analysis

The expression of genes is stochastic in a cell; expression values thus observed are quite heterogeneous at the individual level among seemingly similar cells. The DGE analysis helps to understand the innate cellular processes and stochasticity of gene expressions (McDavid et al., 2013). The problem faced in DGE analysis is identifying genes that are largely expressed in a group of cells without any or no preliminary information of primary cell subtypes (Stegle et al., 2015). Additionally, gene expressions in individual cells show multimodality (Kippner et al., 2014). As expression variability of genes between cells of the same type indicates transcriptional heterogeneity (Johnson et al., 2015; Angermueller et al., 2016), it needs robust computational approaches to detect the true heterogeneity. In addition to multimodality, the sparsity due to—but not limited to—dropout events brings irregularities in the data, consequent of which the differential genes are difficult to detect. Various parametric as well as non-parametric approaches like Single-cell Differential Expression, Model-based Analysis of Single-cell Transcriptome (MAST), D3E, scDD, SigEMD, and DEsingle (Kharchenko et al., 2014; Finak et al., 2015; Delmans and Hemberg, 2016; Korthauer et al., 2016; Miao et al., 2018; Wang and Nabavi, 2018) have been developed/proposed for the DGE analysis in the SC-RNA-seq data. However, these tools try to manage either the gene dropouts or multimodality (Wang et al., 2019). For the subtle DGE analysis, these two crucial challenges need to be taken care of together.

### Cluster Analysis

Cluster analysis of SC-RNA-seq data is required to identify both known and unknown rare cell types (Menon, 2018). Along with the technical dropout events, the cells show a huge variation in gene expression levels even from the same set. As mentioned above, SC-RNA-seq suffers from massive inflation of zeros. There are three reasons for the observation of zeros in data: (1) the transcript was absent explicitly, hence a “true zero”; (2) the depth of sequencing was very low, and the transcript was present but not accounted for; and (3) at the time of library preparation, the transcript could not be captured or failed to amplify. The measurements from the latter two are considered to be the “false zeros.” The concentration of too many zeros in the data brings in irregularities. These technical and biological factors lead to significant noise, due to which cluster analysis becomes challenging. For this, methods like Seurat, DropClust, and SCANPY (Satija et al., 2015; Ntranos et al., 2016; Yip et al., 2017; Sinha et al., 2018) have been proposed for clustering of SCs. There are certain limitations associated with these as well. Seurat and SCANPY work well with large datasets but underperforms when the dataset is smaller (Kiselev et al., 2019). The anticipated complexity in data and the rate of generation of SC data will be a challenge for all these tools. UMAP is yet another method for cluster identification of SC-RNA-seq data; however, as UMAP tends to preserve the local-topological structure, it is rather difficult to establish a relationship between clusters when the underlying cell subtypes are unknown.

In addition to the sparsity in data, SC-RNA-seq data suffer from a huge level of noise from faulty experimental designs usually referred to as “batch-effects.” The noise in the data may contribute to the overfitting of the data. The overfitting can be avoided using regularization. Regularization is a process of restricting or reducing the features at the time of modeling.

So far, the clustering methods cluster the cells as per the transcription similarity, but the biological annotation of cell clusters remains a challenge. A possible solution could come from the generation of the data itself, as the more data are accumulated, the more can unknown clusters be matched with the previously known clusters. Another popular approach for cluster annotation is to use Gene Ontology (GO) analysis of the marker genes (Ashburner et al., 2000).

### Single-Cell Spatial Transcriptomics and RNA Velocity

Spatial transcriptomics (ST) gives measurement of gene expression changes with reference to geographical coordinates of the cells in tissues. It allows measurements of the transcripts with an advantage of conserving the spatial information, providing an additional analytical edge (Burgess, 2019). ST conform to *in situ* methods like seqFISH (Shah et al., 2016), seqFISH+ (Eng et al., 2019), FISSEQ (Fluorescence *in situ* Sequence) (Lee et al., 2015), MERFISH (Chen et al., 2015), and SC-RNA-seq-based methods like slide-seq (Rodriques et al., 2019) and Niche-seq (Medaglia et al., 2017). *In situ* labeling of the transcripts in tissues is advantageous for visualizing the location; however, a chance of molecular overcrowding results in fluorescence signal overlap. This overcrowding can be overcome by using SC spatial RNA-seq; however, the dissociation of cells prior to sequencing makes it difficult to link the transcriptomes back to their original locations (Burgess, 2019). These complementary strengths and limitations make it necessary to integrate the datasets generated by each technology.

In ST, a pair of images are generated, one containing whole tissue with fairly visible spots and the other having clearly visible fluorescence array spots (Wong et al., 2018). To leverage the ST, the image data from ST need to be integrated with the SC-RNA-seq data. As the principle challenges in both ST and SC-RNA-seq are the sparsity of the data and noise from technical and biological sources, an accurate data normalization and transformation is necessary before any downstream analysis (Wagner et al., 2016). Few tools have been developed to determine the cell types with respect to their spatial identities (Edsgärd et al., 2018; Svensson et al., 2018; Dries et al., 2019; Queen et al., 2019). These tools lack interactive processing of images and fails in providing a comprehensive three-dimensional view of the tissue. Recently, STUtility (Bergenstråhle et al., 2020b)—an R package using non-negative matrix factorization (NMF) for reducing the dimensions, spatial correlation (based on Pearson correlation), and K-means clustering—was found capable of providing a holistic view of the expression in tissues. SpatialCPie (Bergenstråhle et al., 2020a) is another easy-to-use R package that uses clustering at various resolutions to interactively uncover the gene expression patterns. Elosua-Bayes et al. (2021) developed SPOTlight, which uses NMF along with non-negative least squares (NNLS). NMF helps in dimensional reduction, followed by selection of marker genes using seurat package and then using NNLS to deconvolute each captured location (Elosua-Bayes et al., 2021).

The SC-RNA measurements have advanced our understanding of the intrinsic cellular functionalities; however, the destruction of cells in the process ceases the possibility of further resampling for an additional transcriptional state analysis. A new methodology, RNA velocity, is capable of deducing the future transcriptional state of a cell (La Manno et al., 2018). The idea behind the study is that the transcriptional upregulation of gene at a particular stage leads to the short-spanned abundance of unspliced transcripts. Similarly, the downregulation of the gene at a point of time results in a decrease of spliced transcripts. The ratio of this variation between unspliced and spliced transcripts is used to estimate the future state of a cell.

### Single-Cell Multi-omics and Data Integration

Biological activities in cells are perplexing, and the measurements of these processes show contrasting variation at temporal and histological levels. To comprehensively understand the intricate biological process of cells and organisms, it is necessary to investigate them at a multi-omics scale. Contingent upon the research question, SC experiments have flexed its reach to variety of layers, the majority of which include the following: (1) SCI-seq for Single-cell Genome Sequencing (Vitak et al., 2017), (2) scBS-seq for Single-cell DNA methylation (Smallwood et al., 2014), (3) scATAC-seq for Single-cell chromatin accessibility (Buenrostro et al., 2015), (4) CITE-seq for cell Surface Proteins (Stoeckius et al., 2017), (5) scCHIP-seq for Histone Modifications (Gomez et al., 2013), and (6) scGESTALT (Frieda et al., 2017) and MEMOIR (Raj et al., 2018) for chromosomal conformation. A universal challenge for all the SC technologies is that the measurements from a very low starting material led to generation of highly sparse and extremely noisy data. Hence, the integration of this data requires a statistically sound and robust computational framework. A primary challenge thereof remains to find an empirical strategy to normalize, batch-effect correction and linking the data from different sources so that the biological meaning and inference remain uncompromised.

For the integration and analysis of the SC multi-omics data, several methods developed for the variety of SC-mono-omics data have been fused or extended further to fulfill the requirement. However, each tool follows a different strategy for the analysis, which can be categorized as follows: (1) correlation and unsupervised cluster analysis; (2) data integration of different samples from a single measurement type and a single experiment type, e.g., SC-RNA-seq; (3) analysis and integration of data from different experiments and a single measurement type across different samples, e.g., sc-Spatial Transcriptomics; (4) integration of data from SC population, with more than one measurement type, different samples, and a single experiment; and (5) integration of data across multiple cells, multiple experiments, and multiple measurement types, e.g., combination of the SC-RNA-seq, scATAC, scCHIP-seq, CITE-seq, etc., of different cells collected at different time points (Stuart et al., 2019; Lähnemann et al., 2020; Lee et al., 2020).

Computational methods and tools for integration of biological data are evolving gradually. A number of techniques have been developed that have been discussed in section “Cluster Analysis.” Seurat (Butler et al., 2018) is currently at the top of integrative analysis of SC multi-omics data, integrating the datasets based on the second principle. Along with Seurat, mutual nearest neighbor (MNN)-based method (Haghverdi et al., 2018) has been exploited to analyze the data combined on the basis of the second category. For the fourth category, analytical methods developed for bulk cellular analysis like MOFA (Argelaguet et al., 2018), MINT (Rohart et al., 2017a), mixOmics (Rohart et al., 2017b), and DIABLO (Singh et al., 2019) are being utilized. Cardelino (McCarthy et al., 2018), MATCHER (Welch et al., 2017), and cloealign (Campbell et al., 2019) are currently the tools used for integrative analysis under the fourth category. To our knowledge, there are no tools available for the last category.

### Big Data Pertaining to Single-Cell RNA Sequencing

**FIGURE 2:**
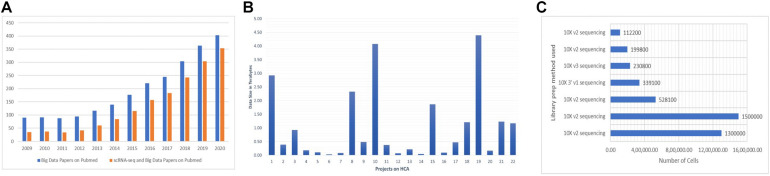
**(A)** There is a steep rise every year for the publications of studies addressing the big data and SC-RNA-seq. For big data papers on PubMed, we used the query “*[big data (All Fields) AND MapReduce (All Fields) AND Hadoop (All fields)]*.” For SC-RNA-seq and big data papers on PubMed, we used “*[(scRNA-seq OR Big Data) OR (Single-cell AND big data)]*.” **(B,C)** Numbers were collected from the Human Cell Atlas Data portal of some exemplary projects.

The data-intensive scientific discoveries rely on three paradigms—theory, experimentation, and simulation modeling (Tolle et al., 2011). As big data is described with three characteristics (volume, velocity, and variety) (Stephens et al., 2015; Adil et al., 2016), data generated by SC-RNA-seq are tantamount to these three quantitative characteristics (Ivanov et al., 2013). With the introduction of new methods in microfluidics (Zare and Kim, 2010), combinatorial indexing procedures (Fan et al., 2015), and rapid drop in the sequencing cost, SC assay profiling has widely become a routine practice among biologists for analyzing millions of cells in hours, paving the way for the accumulation of a large amount of data. The most popular next-generation sequencing platform, Illumina HiSeq, results in the accumulation of around 100 gigabytes of raw RNA-seq data per study. It usually takes hours to align these raw data to their reference genome. SC experiments generating petabytes of data on a variety of layers contribute to the big data paradigm. A human genome has 20,000–25,000 genes composed of 3 million base pairs, totaling to 100 gigabytes of data, equivalent to 102,400 photos^1^; it is expected that more or less “25 petabytes” of genomic data will be generated annually around the globe by the year 2030 (Khoury et al., 2020). It is anticipated that human genomic data can potentially overtake the data produced by online social networks (Check Hayden, 2015). The Human Cell Atlas (HCA)—a project to prepare a reference map of each cell in the human body at various stages, will accumulate a massive amount of data by the end of its completion (Regev et al., 2017). There is a need for comprehensive integration of big data and SC-RNA-seq technologies. A large number of publications on SC-RNA and big data have emerged lately (Figure 2A). The datasets of 4.5 million cells are already published in Data^2^, the largest of which contains more than 1.5 million CD34^+^ hematopoietic cells of human bone marrow (Setty et al., 2019) and 1.3 million transcriptomes of mouse brain cells (Figures 2B,C).

Consequently, the data acquired from these experiments constitute a data revolution in the field of SC biology (Lähnemann et al., 2019). As SC-RNA-seq data have a greater potential of uncovering the hidden patterns at the molecular level, the data pertaining to it thus require an extremely parallel, scalable, and statistically sound computational framework as its handling tools. Big data technologies like Apache’s Hadoop (Taylor, 2010; O’Driscoll et al., 2013) and Spark (Zaharia et al., 2016; Guo et al., 2018) embody the required computational parallelism and data distribution mechanisms. Hadoop uses MapReduce technology for parallel and scalable processing (Dean and Ghemawat, 2008) to disintegrate the larger problems into smaller subproblems on a distributed file system called Hadoop Distributed File System (HDFS). Incorporating big data technologies in the analysis of rapidly increasing SC genomics data will help in transforming and processing it with limitless scalability and fault tolerance at a very low cost.

## Conclusion and Future Perspective

As a consequence of meager RNA capture rate, low starting materials, and challenging experimental protocols, the SC-RNA-seq faces computational and analytical challenges. The noise and sparsity due to the technical (dropout events) and biological factors make the downstream analysis of SC-RNA-seq data a complicated task. Additionally, the rapidity in the development of new and exciting experimental methods for SC-RNA-seq is paving the way for a large accumulation of data. This large agglomeration of data is nothing but the genomic face of “big data.” These two challenges together give rise to a new paradigm of Big Single-Cell Data Science. Although a plethora of algorithms and computational tools have already been developed, it is essential to address these challenges collectively and produce a robust, accurate, parallel, and scalable framework.

## Author Contributions

MA and ATJ conceived the idea, edited the manuscript, and contributed to the compilation of data for designing of figures. AA, VK, and ATJ contributed to the writing of the manuscript. All authors contributed to the article and approved the submitted version.

## Conflict of Interest

The authors declare that the research was conducted in the absence of any commercial or financial relationships that could be construed as a potential conflict of interest.

## Footnotes

1 https://www.experfy.com/blog/intersection-genomics-big-data

2 https://data.humancellatlas.org/
